# Case Report: Area of focus clinical presentation and KMT2D gene mutation at the c.15535C>T site in a case of Kabuki syndrome

**DOI:** 10.3389/fgene.2025.1523228

**Published:** 2025-03-07

**Authors:** Wen Li, Mengjie Lin, Jinwei Dao, Li Shi, Wei Yi, Jia Lei, Yaxian Song, Jiaolou Dong, Meiwei Zhao, Yushan Xu, Lulu Chen

**Affiliations:** ^1^ Department of Endocrinology, People’s Hospital of DeHong, Dehong, Yunnan, China; ^2^ Dehong Biomedical Engineering Research Center, Dehong Teachers’College, Dehong, Yunnan, China; ^3^ Department of Endocrinology, The Frist Affiliated Hospital of Kunming Medical University, Kunming, China; ^4^ Department of Clinical Laboratory, People’s Hospital of DeHong, Dehong, Yunnan, China; ^5^ Department of Science and Education, People’s Hospital of DeHong, Dehong, Yunnan, China; ^6^ Department of Ultrasonography, People’s Hospital of DeHong, Dehong, Yunnan, China; ^7^ Department of Internal Medicine, People’s Hospital of Longchuan DeHong, Dehong, Yunnan, China; ^8^ Department of Endocrinology, Union Hospital, Tongji Medical College, Huazhong University of Science and Technology, Wuhan, China

**Keywords:** Kabuki syndrome, male breast development, KMT2D gene, mutation, clinical presentation

## Abstract

**Background:**

Kabuki syndrome (KS) is a rare autosomal dominant genetic disorder. The full understanding of KS remains elusive due to the heterogeneity of gene mutations, clinical phenotypes, and the associations and mechanisms linking genotypes to phenotypes. This study reports on a 16-year-old male patient diagnosed with type I Kabuki syndrome following the identification of a *de novo* mutation, c.15535C>T (p.Arg5179Cys), in the *KMT2D* gene.

**Case Report:**

A 16-year-old male presented with bilateral breast enlargement persisting for over 1 month. Historically, the patient exhibited intellectual disability. Both parents are healthy with no similar family history. The patient’s father had a history of heroin use for 8 years prior to the patient’s birth. On examination, the patient had unclear speech and slow speech rate, with diminished reading comprehension and calculation abilities. Characteristic facial features of KS were noted. Breast development was observed (Tanner stage II on the right and III on the left), with pain upon deep palpation of the left nipple. Molecular genetic testing identified a heterozygous missense mutation, c.15535C>T (p.Arg5179Cys), in the*KMT2D*gene, confirming the diagnosis of type I Kabuki syndrome.

**Discussion:**

KS is characterized by distinctive facial features: arched eyebrows, eversion of the eyelids, long palpebral fissures, a short nasal septum, a flat nasal tip, auricular deformities, a small mandible, a high palatal arch, or cleft palate. The patient exhibited a heterozygous missense mutation in the coding region of the *KMT2D* gene, identified as a *de novo* mutation. Currently, KS management primarily involves symptomatic and rehabilitative therapies.

## 1 Introduction

Kabuki syndrome (KS) has an incidence of approximately 1:30,000 (Del Cerro et al., 2020) and was first described by Niikawa et al. (1981) and Kuroki et al. (1981) in 1981. Patients with KS often present with distinctive facial features resembling those of Kabuki actors, . KS is a rare autosomal dominant genetic disorder caused by mutations in the lysine (K)-specific methyltransferase 2D (*KMT2D*) gene or the X-linked lysine-specific demethylase 6A (*UTX/KDM6A*) gene. The first case of KS in China was reported in 1998 (Jiao et al., 1998). The typical clinical manifestations of KS include distinctive facial features: arched eyebrows, eyelid eversion, long palpebral fissures, short nasal septum, flat nasal tip, auricular deformities, small mandible, high palatal arch, or cleft palate. These features are crucial for distinguishing KS from other multiple malformation syndromes (Xu, 2007). Other common manifestations include short stature, brachydactyly, joint laxity, hypotonia, skeletal anomalies, intellectual disability, speech impairments, hearing loss, inner ear malformations, congenital heart defects, urogenital malformations, dental anomalies, epilepsy (Li, 2018), and dermatoglyphic anomalies, with persistent fetal fingertip pads being characteristic symptoms of KS. Endocrine abnormalities such as precocious puberty and isolated premature thelarche, athelia, polythelia, hypoglycemia, hormonal imbalances are more common in patients with *KMT2D* mutations. The etiology of these features has not been definitively established, but Pescowitz et al. suggest it may be due to premature activation of hypothalamic gonadotropin-releasing hormone (LHRH) neurons. Male breast development has not been previously reported. This study reports on a 16-year-old male patient found to have a mutation in the *KMT2D* gene, c.15535C>T (p.Arg5179Cys), leading to a diagnosis of type I Kabuki syndrome.

## 2 Case description

### 2.1 Subject information

**FIGURE 1:**
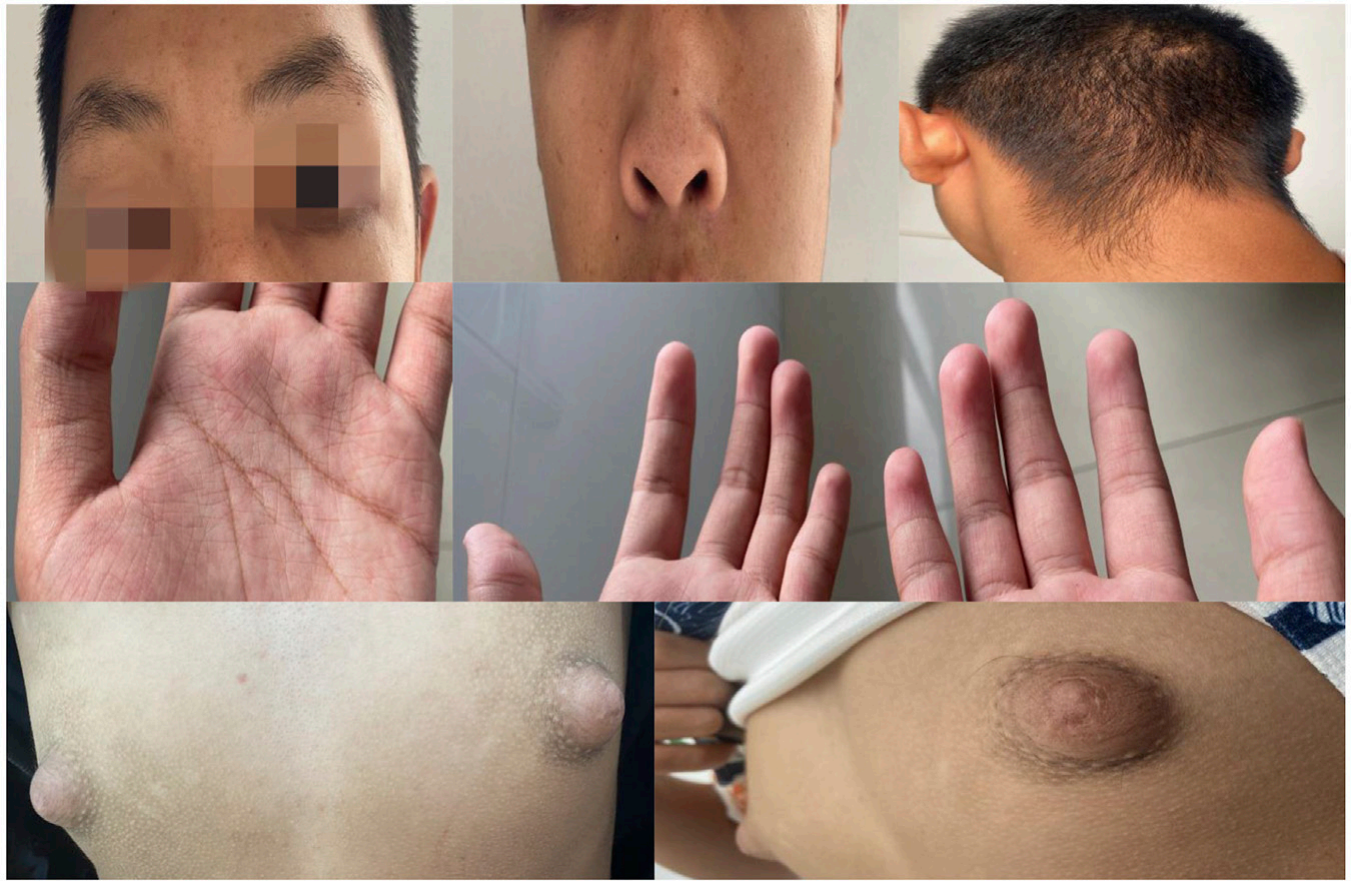
Distinctive Facial and Physical Features: Arched eyebrows with sparse outer thirds; long palpebral fissures with mild eversion of the lower eyelids; a short nose with a flat nasal tip; low posterior hairline. Dermatoglyphic Anomalies: Increased number of palmar lines, fine and multiple creases; visible fetal fingertip pads on all digits; breast hypertrophy.

The patient, a 16-year-old male, was delivered at home with no history of asphyxia or birth trauma. Birth weight was 2.8 kg. The mother (G2P2) underwent regular prenatal check-ups, remained healthy during pregnancy, and was not of advanced maternal age (25 years), with no significant medication history. Both the father and the patient’s older sister are in good health. The child was breastfed for the first year of life, with timely introduction of complementary foods. The child could stand independently at 1 year but did not begin walking independently until the age of two and a half, with an unsteady gait. At 3 years old, the child could only speak simple repeated words like “Papa” and “Mama,” unable to form complete sentences or engage in communication effectively. He often faced bullying during play and preferred solitary play. At the age of three, parents noticed delays in speech, walking, and height development compared to peers and sought medical attention, resulting in a diagnosis of developmental delay. A laryngeal examination was recommended but not performed. There is no history of epilepsy, hypoglycemia, constipation, celiac disease, malnutrition, or heart defects. The child has not taken any medication for chronic diseases and has been vaccinated as per the schedule. The child began receiving compulsory education on time but transferred to a special school in second grade due to poor academic performance. Height was below average for their age group. Height growth accelerated at the age of 15, increasing by 6 cm last year. Secondary sexual characteristics (voice change, ejaculation, or clear signs of erection) were not observed. Appetite remains good, with no nausea, vomiting, or diarrhea. Weight increased by 3 kg over the past year. He was admitted after noticing a bilateral breast hypertrophy for over a month. The enlargement was more pronounced on the left side and was painful upon palpation. No nipple discharge was observed. The patient had not used anti-androgens, estrogens, diuretics, or glucocorticoids. Initial evaluation by the general surgery department at our hospital, followed by a color ultrasound of the breasts, indicated male breast development. The parents are nonconsanguineous, and there is no similar family history. The patient’s father had an 8-year history of heroin use prior to the patient’s birth. Physical examination revealed unclear and slow speech, moderate intellectual disability (Using the Wechsler Intelligence Scale for Children, the child scored an IQ of 45),and reduced reading comprehension and calculation abilities. Distinctive facial and physical features (see Figure 1) included a flat expression, small mandible, arched eyebrows with sparse outer thirds, high palatal arch, ptosis of the upper eyelids, long palpebral fissures with mild eversion of the lower eyelids, a short nose with a flat tip and flared nostrils, a thin upper lip and thick, indented lower lip. Fetal fingertip pads were visible on all digits; the palmar lines were numerous, fine, and had many folds and transverse palmar creases. The fifth digits of both hands were short and bent outward. Bilateral breast development was noted (Tanner stage II on the right and III on the left), with pain on deep palpation of the left nipple. There was no axillary hair growth, and the posterior hairline was low. Pubic hair was sparse, curly, and limited to the base of the penis; the penis measured 2.2 cm, with right testicular volume of 3 mL and left of 4 mL. The patient’s developmental timeline in Table 1.

**TABLE 1:** The patient’s developmental timeline.

Age	Height (length) (cm)	Weight (kg)	Physical development	Language development
Birth	50	2.8	He Can crying, no feeding difficulties	
1 years old	62	7.2	stand independently	He could not speak any words
1–2years	68	8.4	He can not walking independently	He could not speak any words
2 years and a half	76	10.6	He can walking independently with an unsteady gait	He could not speak any words
3 years old	79.2	11.2	Parents noticed delays in speech, walking, and height development compared to peers and sought medical attention, resulting in a diagnosis of developmental delay. A laryngeal examination was recommended but not performed. Intelligence quotient of 45	He could only speak simple repeated words, unable to form complete sentences or engage in communication effectively. He often faced bullying during play and preferred solitary play
7 years old	113.5	20.8	He also delays in speech, walking, and height development compared to peers,but Parents did not sought medical attention	The child began receiving compulsory education on time but transferred to a special school in second grade due to poor academic performance
11 years old	135.3	45.3	Secondary sexual characteristics were not observed	He unable to form complete sentences or engage in communication effectively
15 years old	172.5	59.8	Obvious growth in height, Secondary sexual characteristics were not observed	He unable to form complete sentences or engage in communication effectively
16 years old	178.0	63	He was admitted after noticing a bilateral breast hypertrophy for over a month.Secondary sexual characteristics were observed, Bilateral breast development was noted (Tanner stage II on the right and III on the left), with pain on deep palpation of the left nipple. There was no axillary hair growth, and the posterior hairline was low. Pubic hair was sparse, curly, and limited to the base of the penis; the penis measured 2.2 cm, with right testicular volume of 3 mL and left of 4 mL	He unable to form complete sentences or engage in communication effectively

### 2.2 Supplementary examinations

Breast ultrasound revealed male breast development (right 4.5 cm × 1.1 cm, left 5.3 cm × 1.6 cm). Electrocardiogram, thyroid ultrasound, cardiac echocardiogram, urinary system ultrasound, MRI of the brain and pituitary, X-rays of the pelvis and spine showed no abnormalities; Bone age assessment corresponded to approximately 17 years in a male. Bone density indicated reduced bone mass; chromosomal karyotype analysis revealed a 46,XY karyotype. Blood test results are presented in Table 2.

**TABLE 2:** Laboratory test results.

Item	Results	References range
Complete Blood Count		
White Blood Cell Count (×10^9^/L)	5.53	3.5–9.5
Red Blood Cell Count (×10^12^/L)	3.97	3.5–5.5
Platelet Count (×10^9^/L)	244	125–350
Hemoglobin (g/L)	159 g/L	115–150
Liver and Kidney Function		
Alanine Aminotransferase (U/L)	17	7–40
Aspartate Aminotransferase (U/L)	16	13–35
Albumin (g/L)	50.6	65–85
Urea (mmol/L)	3.7	2.6–7.5
Creatinine (μmol/L)	78	41–73
Lipid and Glucose Profile		
Triglycerides (mmol/L)	0.61	<1.7
Total Cholesterol (mmol/L)	2.42	<5.2
High-Density Lipoprotein (mmol/L)	1.79	≥1.0
Low-Density Lipoprotein (mmol/L)	0.44	<3.4
Glucose (mmol/L)	4.75	3.9–6.1
Pituitary-Thyroid Axis Function		
Thyroid-Stimulating Hormone (mIU/L)	3.53	0.27–4.2
Pituitary-Adrenal Axis Function		
Adrenocorticotropic Hormone (pg/mL)	22.0	6–48
Cortisol (ng/mL)	97.89	72.6–322.8
Pituitary-Gonadal Axis Function		
Follicle-Stimulating Hormone (IU/L)	5.97	(≤12.4)
Luteinizing Hormone (U/L)	5.39	1.7–8.6
Estradiol (pmol/L)	35.2	94.8–223
Prolactin (mIU/L)	167	86–324
Testosterone (nmol/L)	18.88	0.98–38.5
Parathyroid		
Parathyroid Hormone (pg/mL)	39.67	15–65
Blood Calcium (mmol/L)	2.57	2.1–2.55

Gene	Chromosome location	Variant information	Zygosity type	Disease name	Inheritance pattern	Source of mutation	Variant classification
*KMT2D* Gene Mutation: c.15535C>T (p.Arg5179Cys)							
*KMT2D*	Chr12:49,420,214	NM_003482.3:c.15535C>T (p.Arg5179Cys)	Heterozygous	Kabuki Syndrome Type 1 [MIM:147920]	AD	*De novo* Mutation	Pathogenic Variant
*TAF1* Gene Mutation: c.16G>T (p.Asp6Tyr)							
*TAF1*	chrX:70,586,180	NM_004606.4:c.16G>T (p.Asp6Tyr)	Hemizygous	X-linked Intellectual Developmental Disorder Type 33 [MIM:300966]	XLR	Mother	Variant of Uncertain Significance

### 2.3 Genetic testing methods

Genetic testing was performed using next-generation sequencing data for single nucleotide variations, small segment insertions and deletions, and large segment copy number variations. Real-time quantitative PCR and Western blot analyses were employed to evaluate the transcriptional and protein expression levels of the *KMT2D* gene (also called *MLL2* gene, OMMI:147920) respectively.

### 2.4 Genetic testing results

A pathogenic variant that explains the patient’s phenotype and another variant correlating with the patient’s main clinical features were detected, though the latter’s clinical significance remains uncertain. The results indicated the presence of a heterozygous missense mutation in the coding region of the *KMT2D* gene: c.15535C>T (p.Arg5179Cys).

**FIGURE 2:**
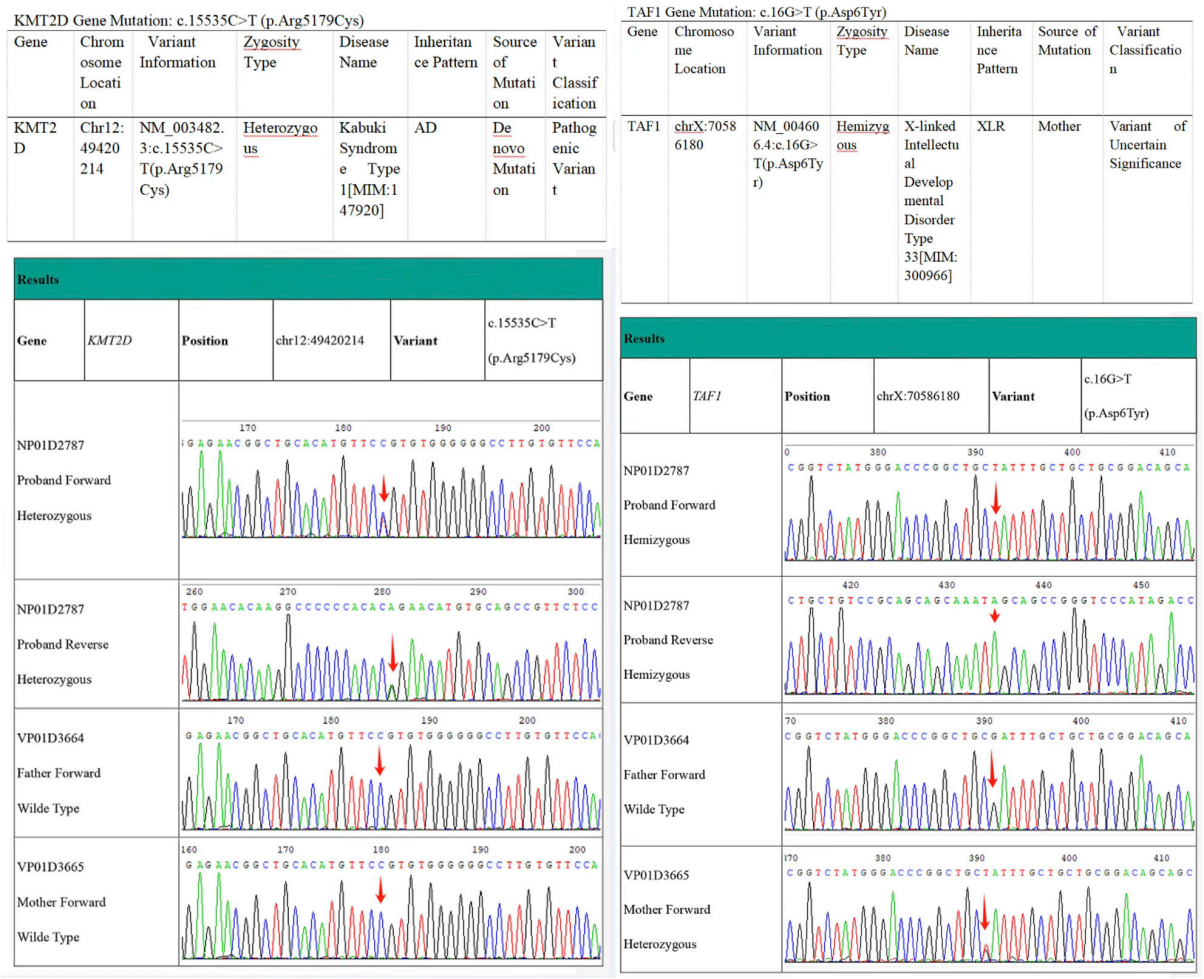
Electropherogram of *KMT2D* Sequencing/Electropherogram of TAF1 Sequencing. Comparison of the 15535th base with the patient’s parents reveals that the cytosine (C) in the patient has mutated to thymine (T).

Additionally, genetic testing identified a hemizygous missense mutation in the *TAF1*gene: c.16G>T (p.Asp6Tyr). This variant can cause X-linked syndromic intellectual developmental disorder 33 (MRXS33) [MIM:300966], inherited in an X-linked manner. The c.16G>T (p.Asp6Tyr) variant, inherited from the patient’s mother, represents a missense mutation in the *TAF1* gene coding region. This variant has not been reported in large-scale population frequency databases or literature. Based on available evidence, this variant is classified as a variant of uncertain clinical significance (see Figure 2). No clinical manifestations related to TAF1 gene mutations were observed in the patient.

### 2.5 Detection of *KMT2D* gene transcriptional and protein expression levels

**FIGURE 3:**
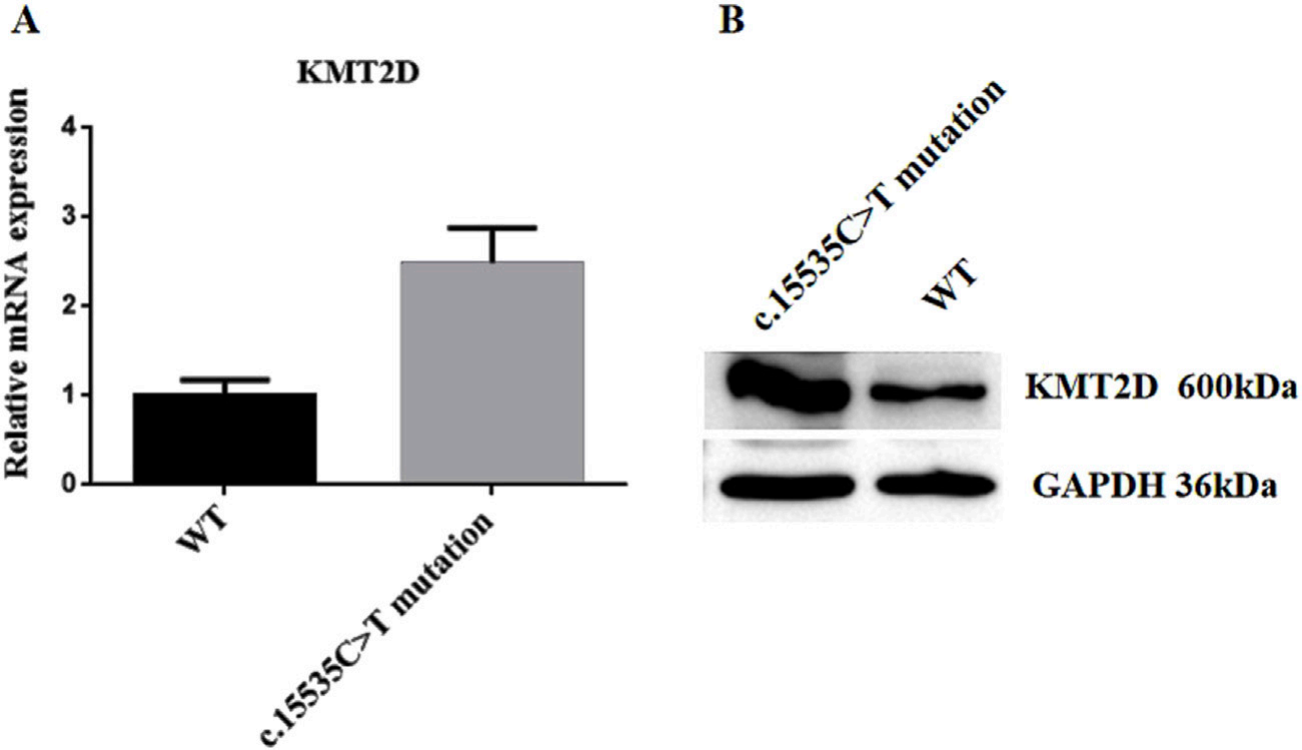
*KMT2D* Expression Analysis. The back skin fibroblasts of the patient were isolated and cultured to the third generation, and the normal human fibroblasts (BJ cell No. FH0191) were used as the control group. Real-time fluorescence quantitative PCR and protein Western blot detection were performed. **(A)** Real-Time Quantitative PCR Analysis. **(B)** Western blot Analysis. c.15535C>T mutation: Proband’s fibroblasts; WT (Wild Type): Normal fibroblasts.

To validate the expression levels of the *KMT2D* gene at both transcriptional and protein levels in the proband, skin-derived fibroblasts were isolated and cultured in primary cell culture, with normal fibroblasts serving as controls. Real-time quantitative PCR results indicated a higher expression trend of the *KMT2D* gene in the proband’s cells compared to controls. Western blot analysis (SDS-PAGE) also showed increased protein expression levels of *KMT2D* in the proband’s cells compared to controls, consistent with the transcriptional level results. These findings suggest that the *KMT2D* gene harboring the c.15535C>T (p.Arg5179Cys) mutation exhibits upregulated expression at both transcriptional and protein levels (see Figure 3).

### 2.6 Diagnosis

The patient has been diagnosed with Type I KS.

### 2.7 Treatment

There is no specific treatment for KS; management primarily involves symptomatic and rehabilitative therapies, with systematic follow-up of growth and development indices and symptomatic treatment tailored to the clinical manifestations at different stages. The patient’s gonadal development is slightly delayed, but pituitary-gonadal axis hormones are currently normal, and he is still in puberty, thus continuing observation is recommended. The development of male breast tissue will continue to be monitored, and post-puberty, surgical intervention may be considered if aesthetically or psychologically necessary (Thiruchelvam et al., 2016). By evaluating the pathogenic gene causing KS, targeted therapy may be considered. Gene therapy or histone methylation-modulating molecules for Kabuki Syndrome (KS) remain in the research stage. Early identification, diagnosis, and personalized treatment for KS can improve prognosis. Genetic counseling can reduce the birth of children with genetic disorders and effectively lower birth defects.

The parents expressed relief that their child received an accurate diagnosis and treatment, resolving a long-standing doubt about why their child differed from peers. They expressed gratitude to the doctors and agreed to continue monitoring for breast tissue overgrowth until 18 years old, consenting to surgery if necessary. Moving forward, they plan to provide more care and focus on their child’s psychological health and nutrition.

## 3 Discussion

This article reports on a 16-year-old case of KS, in which comprehensive exome sequencing revealed a heterozygous missense mutation in the coding region of the *KMT2D*gene, c.15535C>T (p.Arg5179Cys), ultimately leading to a diagnosis of Type I Kabuki Syndrome. There is a heterozygous missense mutation in the coding region of the *KMT2D*gene:c.15535C>T (p.Arg5179Cys). At the 15535th base of the *KMT2D* gene, a cytosine (C) was replaced by a thymine (T), resulting in the substitution of the amino acid at position 5,179 from arginine to cysteine, altering the amino acid’s polarity. This mutation was not detected in peripheral blood samples from the patient’s parents, suggesting it is a *de novo* mutation. The mutation is extremely rare, with no reports in HGMD databases. Base on current evidence, this variant is classified as pathogenic, and the c.15535C>T (p.Arg5179Cys) mutation is likely the cause of the patient’s condition (see Figure 2). The particularity of this case lies in the fact that molecular genetic testing found a mutation at the locus of *KMT2D* gene C.15535C>T (P.Arg5179cys), which is a new mutation and has not been reported before. In addition, the patient presented with bilateral breast enlargement, and the father had a history of drug use for 8 years prior to birth. In 2010, Ng et al. (2010) reported that the *KMT2D* gene is the primary causative gene for Kabuki Syndrome Type I, accounting for 56%–75% of KS cases, characterized by autosomal dominant inheritance. Additionally, heterozygous deletions in the Kabuki Syndrome due to a *KDM6A* gene mutation (Bögershausen and Wollnik, 2013),Currently, Detailed distinctive clinical pictures have not yet been fully characterized, But,*KDM6A* mutations are typically associated with more severe phenotypes, including higher risks of epilepsy, developmental delays, and feeding difficulties. Dai Xiaowei et al. reported a case of a 3-year-old child, who presented with developmental delays, found to have a heterozygous mutation in the *MLL2* gene coding region: c.2546 > T (p.Ser849Leu), leading to KS (Dai et al., 2018). Lu Huimin et al. performed genetic testing on a child with characteristic facial features, developmental delays, and cardiovascular abnormalities, identifying a frameshift mutation in the *KMT2D* gene, c.16028delC (p.Pro5343LeufsTer13) (Lu, 2022). Xin et al. (2018) detected two novel mutations in the *KMT2D* gene (c.5235delA, p.(A1746Lfs39) and c.7048G>A, p.(Q2350)) in two patients, confirmed by Sanger sequencing-based family pedigree analysis. These 62 patients with the Kabuki syndrome were collected in a collaborative study among 33 institutions and analyzed clinically, Many other inconsistent anomalies were observed. Important among them were early breast development in infant girls (23%), (Niikawa et al., 1988). Wu et al. described a 10-month-old female infant diagnosed with KS due to a c.12697C>T mutation, presenting developmental delays and early breast development (Wu and Su, 2017).The clinical manifestations in this study align with previous literature, such as developmental delays and unique facial features. Although rare breast tissue overgrowth was observed, the patient had no significant life-threatening organ malformations. The comparison between the performance of this patient and the previous literature are shown in Table 3. Barry et al.'s meta-analysis of 1,369 KS cases suggested that *KMT2D* mutations were predominantly truncation mutations, followed by missense mutations (Barry et al., 2022), consistent with this case’s mutation type.

**TABLE 3:** The comparison between the performance of this patient and the previous literature.

Case	Gender	Age at diagnosis (months)	Clinical features	Genotypic features
Dai et al. (2018)	female	3 years and 7 months	Special features: small head circumference, arched eyebrows, outer third of lower eyelid ectropion, flat nose tip, short nasal septum, flaring nostrils, both ears obviously large, mouth half open, long people, the fifth finger of both hands slightly short, the left hand through the palm, short toes, low back hairline, short neck, bone age backward, the great cisterna of the pillow slightly expanded	KMT2D gene:c.2546 > T (p.Ser849Leu)
Lu (2022)	male	7 months	Special features: long eyelid cleft, lower outer 1/3 of the eyelid ectropion, broad forehead, arched eyebrows, broad outer 1/3 sparse, short nasal column, concave nose tip, short mandible, cup-shaped ears. Head MRI showed slight enlargement of bilateral lateral ventricles and thinning of corpus callosum. The ultrasonic electrocardiogram diagnosed congenital heart disease: aortic arch rupture (type A)	KMT2D gene:c.16028delC (p.Pro5343LeufsTer13)
Xin et al. (2018) Patient 1	male	26 months	Special features:long palpebral fissures with lateral eversion of the lower eyelids, arched eyebrows with laterally thinning, depressed nasal tip, lower lip concave, and large ears. Brachydactyly and prominent finger pads. Sacral dimpling intelligence quotient of 45. Abnormal dentitions. His right side had ectopic kidney, and left side was narrow and had cyst, the demarcation between cortex and medulla of both kidneys was not clear	KMT2D gene:c.5235delA heterozygous mutation, p. (A1746Lfs*39)
Xin et al. (2018) Patient 2	male	6 months	Special features:long palpebral fissures with lateral eversion of the lower eyelids, arched eyebrows with laterally thinning eyebrows, depressed nasal tip, large ears, low hairline, and lower lip concave.He also had brachydactyly and prominent finger pads especially for the fifth finger and palm with a straight line across it, and sacral dimpling. Joint hypermobility and hypotonia. Magnetic resonance imaging of brain suggested cerebellar vermis dysplasia, and urological ultrasound indicated incomplete cryptorchidism on the left side	KMT2D gene:c.7048G > A heterozygous mutation, p. (Q2350*)
Wu and Su (2017)	female	10 months	Developmental delay, early breast development, Special features:long eyelid cleft, lower eyelid ectropion, large ear, wide nose bridge, flat nose tip, cleft palate, fetal finger pad, lateral finger curvature, caudal vertebra deformity, hearing impairment	KMT2D gene:c.12697C>T (P.q4233X)
Our patient	male	16 years	developmental delay, Bilateral breast development (Tanner stage II on the right and III on the left), with pain on deep palpation of the left nipple. Special features: small mandible, arched eyebrows with sparse outer thirds, high palatal arch, ptosis of the upper eyelids, long palpebral fissures with mild eversion of the lower eyelids, a short nose with a flat tip and flared nostrils, a thin upper lip and thick, indented lower lip. Fetal fingertip pads were visible on all digits; the palmar lines were numerous, fine, and had many folds and transverse palmar creases. The fifth digits of both hands were short and bent outward	KMT2D gene: c.15535C>T (p.Arg5179Cys)

KS patients often have multiple endocrine system diseases, such as developmental delay, hypoglycemia, hypothyroidism, precocious puberty, and diabetes insipidus. The underlying mechanisms remain unclear but may involve estrogen receptor dysfunction due to *KMT2D* gene mutations. Our study identified a rare heterozygous missense mutation, c.15535C>T (p.Arg5179Cys), in the coding region of the *KMT2D* gene, not reported in large population frequency databases and not detected in the peripheral blood samples of the patient’s parents, indicating a *de novo* mutation.

Not only did we identify the pathogenic gene mutation, but we also validated the expression levels of the *KMT2D* gene at both transcriptional and protein levels in the proband. The results indicated that compared to normal fibroblasts, the proband’s cells showed increased transcriptional expression of the *KMT2D* gene and elevated protein expression, consistent with the transcriptional data. These findings suggest that the *KMT2D* gene, harboring the c.15535C>T (p.Arg5179Cys)mutation, exhibits upregulated expression at both transcriptional and protein levels, confirming that this gene mutation is likely the cause of the patient’s condition. This case further confirms the impact of *KMT2D* gene mutations on protein levels in KS patients, clarifying the diagnosis and enriching the evidence for the gene’s influence. It contributes to future research on gene therapy and epigenetic modifications, enhancing understanding of the relationship between epigenetic changes and human disease development.

Kabuki syndrome is a rare disease, many doctors lack sufficient understanding of this disease, the most prominent feature of this disease is the special face, because there is no thorough treatment method at present, early prevention, early diagnosis and early treatment are very important, which can reduce the burden of patients and their families to the greatest extent, so further epidemiological investigation and research of this disease are needed. Modern molecular biology diagnostic technology, especially second-generation sequencing, is helpful to find the pathogenic genes of complex diseases, so as to make clear diagnosis, judge prognosis, guide treatment, and truly achieve the purpose of eugenics and fertility.

## Data Availability

The original contributions presented in the study are included in the article/supplementary material, further inquiries can be directed to the corresponding authors.
